# Multidimensional Poverty in Rural Mozambique: A New Metric for Evaluating Public Health Interventions

**DOI:** 10.1371/journal.pone.0108654

**Published:** 2014-09-30

**Authors:** Bart Victor, Meridith Blevins, Ann F. Green, Elisée Ndatimana, Lázaro González-Calvo, Edward F. Fischer, Alfredo E. Vergara, Sten H. Vermund, Omo Olupona, Troy D. Moon

**Affiliations:** 1 Owen Graduate School of Management, Vanderbilt University, Nashville, Tennessee, United States of America; 2 Vanderbilt Institute for Global Health, Vanderbilt University, Nashville, Tennessee, United States of America; 3 Department of Biostatistics, Vanderbilt University School of Medicine, Nashville, Tennessee, United States of America; 4 Department of Preventive Medicine, Vanderbilt University School of Medicine, Nashville, Tennessee, United States of America; 5 Department of Pediatrics, Vanderbilt University School of Medicine, Nashville, Tennessee, United States of America; 6 Friends in Global Health, Maputo, Mozambique; 7 Vanderbilt Center for Latin American Studies and Department of Anthropology, Vanderbilt University, Nashville, Tennessee, United States of America; 8 World Vision International, Maputo, Mozambique; University of South Carolina, United States of America

## Abstract

**Background:**

Poverty is a multidimensional phenomenon and unidimensional measurements have proven inadequate to the challenge of assessing its dynamics. Dynamics between poverty and public health intervention is among the most difficult yet important problems faced in development. We sought to demonstrate how multidimensional poverty measures can be utilized in the evaluation of public health interventions; and to create geospatial maps of poverty deprivation to aid implementers in prioritizing program planning.

**Methods:**

Survey teams interviewed a representative sample of 3,749 female heads of household in 259 enumeration areas across Zambézia in August-September 2010. We estimated a multidimensional poverty index, which can be disaggregated into context-specific indicators. We produced an MPI comprised of 3 dimensions and 11 weighted indicators selected from the survey. Households were identified as “poor” if were deprived in >33% of indicators. Our MPI is an adjusted headcount, calculated by multiplying the proportion identified as poor (headcount) and the poverty gap (average deprivation). Geospatial visualizations of poverty deprivation were created as a contextual baseline for future evaluation.

**Results:**

In our rural (96%) and urban (4%) interviewees, the 33% deprivation cut-off suggested 58.2% of households were poor (29.3% of urban vs. 59.5% of rural). Among the poor, households experienced an average deprivation of 46%; thus the MPI/adjusted headcount is 0.27 ( = 0.58×0.46). Of households where a local language was the primary language, 58.6% were considered poor versus Portuguese-speaking households where 73.5% were considered non-poor. Living standard is the dominant deprivation, followed by health, and then education.

**Conclusions:**

Multidimensional poverty measurement can be integrated into program design for public health interventions, and geospatial visualization helps examine the impact of intervention deployment within the context of distinct poverty conditions. Both permit program implementers to focus resources and critically explore linkages between poverty and its social determinants, thus deriving useful findings for evidence-based planning.

## Background

In the last two decades, the world’s governments have generated unprecedented support for a comprehensive list of global development aims, of which the Millennium Development Goals (MDGs) are an integral part. [1]–[4] One of the most significant outcomes of these joint efforts has been the prioritization of poverty reduction at the center of national and international policy agendas. In fact, the first goal of the MDGs is to reduce by half the global proportion of people living in extreme poverty by 2015. Three additional MDGs target public health interventions that reduce child mortality, improve maternal health, and combat HIV/AIDS, malaria and other disease [1]–[4]. Understanding the link between poverty and the public health interventions employed in the developing world is among the most difficult yet important problems faced today [5], [6].

Increasingly the effectiveness of public health interventions is recognized as being closely tied to the impact of other efforts such as economic development, education, agriculture programs and improvements in infrastructure (including water and sanitation), shelter and security. While each intervention area brings an emphasis and focus on distinct needs, there are significant interactions and co-dependencies between areas. For example, it is generally accepted that poverty is closely associated with the availability and quality of health service, but this relationship is far from simple. There is a complex mutual causation: poor health services contribute to poverty, which in turn negatively influences the ability to access and utilize health services. To determine the impact of public health interventions on poverty reduction, it is necessary to define an appropriate framework for poverty measurement. By creating an evaluation paradigm that establishes an index of “poverty” as the primary outcome measure, yet which can be further disaggregated based on the contributions of its defined dimensions such as health, education, and living standard; we get the added value of being able to evaluate each dimension independently while simultaneously learning from the interactions and co-dependencies between areas that subsequently impact the effectiveness of the interventions employed to address them.

Traditional standards for measuring poverty are ever more criticized for their potential to both “mis-measure” and more importantly, misunderstand the true drivers of poverty. [7] Per capita income is a unidimensional measure that categorizes a person as poor if their income falls below a particular “poverty line”, now frequently defined as less than $1.25 a day. [8]–[10] Unidimensional measures have been extensively critiqued in terms of their validity and precision and have proven inadequate to the challenge of assessing the extent and dynamics of poverty in the world. [7] Critique has been mostly directed toward use of unidimensional measurements for national policy and global comparison purposes. However, this argument is perhaps even more compelling when directed at interventions or public health programming aimed to improve well-being. An alternative approach derived from the work of Amartya Sen, introduces the idea that there are multiple dimensions of poverty [9]–[12].

The International Fund for Agriculture Development (IFAD) and the Sustainable Coffee Partnership, for example, have each developed sophisticated multi-criteria models that take into account many dimensions when measuring their intervention’s success or not. [13] This approach for measuring many dimensions is not the same as measuring multidimensionality. In practice, extant models measure many dimensions, but most do not produce an integrated multidimensional model. One notable exception has been the work of the Oxford Poverty and Human Development Initiative (OPHI). [14] Working within the OPHI framework, Alkire and Foster have developed a method for calculating dimensional weights and cut-offs that integrate a number of dimensions into a combined metric [15].

International development organizations, researchers, and an increasing number of national governments are beginning to adopt multidimensional measures of national poverty. [14], [16] The United Nations Development Program’s (UNDP) Human Development Index (HDI) includes not only measures of Gross National Product (GNP) per head, but also incorporates indicators related to education and health in order to produce a composite index. Some program monitoring and evaluation efforts have also begun to incorporate multiple measures.

The three main objectives of this paper are 1) to demonstrate how multidimensional measures of poverty can be utilized in the planning and evaluation of large scale public health and development interventions; 2) to utilize the Alkire and Foster method for multidimensional poverty measurement in Zambézia Province, an extremely rural, infrastructure-depleted region of north-central Mozambique, to establish a baseline poverty index from which future comparisons will be conducted in order to measure the impact of interventions in the province; and 3) to create geospatial maps of poverty deprivation at baseline, providing program implementers a visual needs assessment that characterizes the geographic differences of each target district and aids in prioritizing program planning.

## Methods

### Study Context

In 2009, World Vision International was awarded a United States Agency for International Development (USAID) grant called Strengthening Communities through Integrated Programming (SCIP) for a 5-year multi-sector program aimed at improving the health and livelihoods of children, women, and families in Zambézia Province, Mozambique. Known locally as the *Ogumaniha* project, which means “united for a common purpose” in the local language of Echuabo, SCIP is based on a consortium of five international non-governmental organizations led by World Vision. The broad goals of the 5-year project are to: 1) reduce poverty in Zambézia Province by pursuing the consolidation of an integrated, innovative, and sustainable community-based program in the province; and 2) integrate current and future United States Government (USG) investments in Zambézia Province in the areas of health, HIV/AIDS, water and sanitation, income generation, and institutional capacity building.

In 2012, Mozambique ranked 185 of 187 nations on the UNDP’s HDI, and the gross national income was estimated at US $906 per capita [8], with male and female life expectancies of 47 and 51 years, respectively, in 2009 [17]. Although Mozambique’s health expenditure has risen substantially over the past 10 years, as a proportion of total GDP it was only 6.6% in 2011 (66 USD per capita). [18] Mozambique is one of the sub-Saharan African countries most affected by the HIV/AIDS epidemic, with a national adult HIV prevalence of 11.5% in 2009. [19] Nationally, 12% of children were considered orphans or vulnerable children, and only 43% of households had access to clean drinking water in 2009 [20].

**Figure 1:**
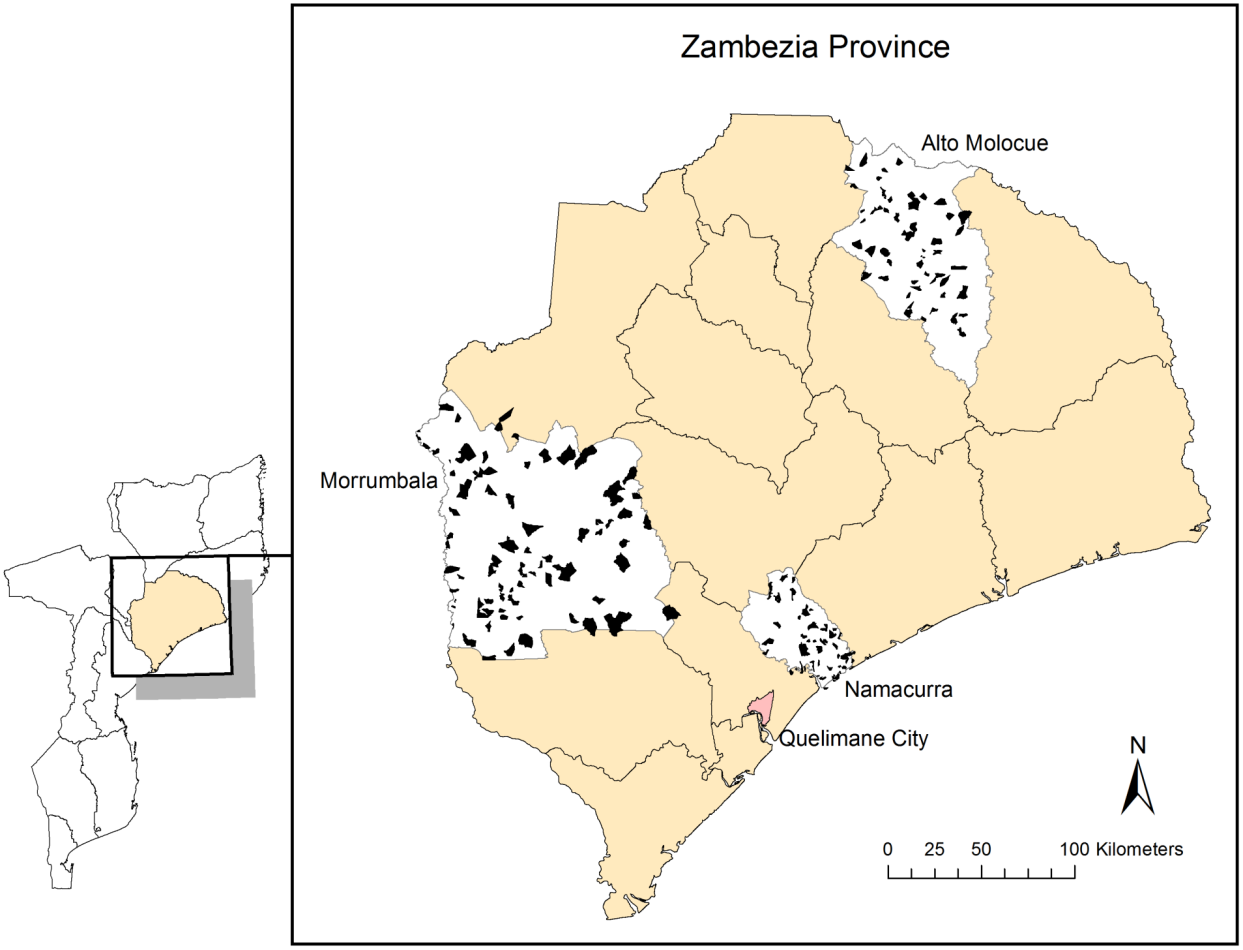
Map of Mozambique, Zambézia Province, with Enumeration Areas Highlighted in Three Focus Districts, Namacurra, Morrumbala, and Alto Molócuè.

The magnitude of poverty is especially evident in Zambézia Province, Mozambique’s second largest province and home to about 4 million persons (Figure 1). [21], [22] While Mozambique ranks among the poorest of the poor nations, Zambézia consistently ranks among Mozambique’s lowest performing provinces with low literacy rates, poor maternal and child health (MCH) indices, high rates of tuberculosis and malaria infections, and high levels of malnutrition. [20], [23], [24] Zambézia Province is overwhelmingly rural and depends almost entirely on subsistence farming and fishing. The province has the highest estimated number of persons living with HIV in the country (∼275,000 or nearly 20% of Mozambique’s HIV-infected population) as of 2009 [19], [25] and yet only 31 of Zambézia’s 214 health facilities provided antiretroviral therapy (ART) as of December 2012. This is partially because Zambézia Province housed much of the armed conflict in Mozambique’s 16-year civil war (1976–1992) and suffered disproportionately in destruction of its healthcare infrastructure [21], [22].

### Study Design

Integral to *Ogumaniha’s* design is a strong monitoring system and project evaluation based on performance indicators agreed upon with USAID and the provincial government. Because the project involves multi-sectoral interventions and an interdisciplinary approach to implementation, the consortium opted for a multidisciplinary evaluation design. A survey instrument used at *Ogumaniha*’*s* initiation (baseline survey in 2010) and at the project’s end (final survey implemented June 2014) was designed based on the human development theory originated by Sen (1999) and further developed by researchers from OPHI. This instrument uses multiple dimensions to measure poverty including health, education, and income; access to goods and services; and self-empowerment. The vision of this pre-post project evaluation is that the information collected can provide a more thorough and holistic measure of the impact of this large-scale, multi-sector intervention on the overall health and well-being of the households in Zambézia Province, more so than analysis of the individual sector specific measures when viewed in isolation.

The *Ogumaniha* survey tool collects information on over 500 variables in 8 dimensions and was developed by a multi-disciplinary team of researchers including staff, faculty, and graduate students from Vanderbilt University and the Universidade Eduardo Mondlane. To design the survey, we used many questions and validated scales from previous national surveys in Mozambique, including various National Institute for Statistics (*Instituto Nacional de Estatísticas* [INE]) surveys focusing on poverty and economic status; and other international surveys such as the Demographic Health Survey (DHS) and the Multiple Indicator Cluster Survey (MICS). The survey was designed to collect household information from the female head of household, defined as the principal wife of the nuclear family (polygamy is common practice), because she is thought to be most familiar with the majority of topics of interest. Survey questions covered household demographics; economic status; health knowledge, attitudes and practices; access to health and HIV-related services and products; access to improved water and sanitation; nutritional intake; and others.

The poverty index used to identify households or areas of poverty in the province following baseline survey data collection, was modeled after the Multidimensional Poverty Index (MPI) founded on the OPHI methodology which calculates the quantity of defined “poor” (the headcount), multiplied by their average amount of deprivation, called the Adjusted Headcount Ratio [15].

### Data Collection

Mobile survey teams conducted interviews with 3,916 (98%) of 3,960 planned female heads of households in 259 Enumeration Areas (EAs) across 14 of Zambézia’s 17 districts. Complete data for analysis was available from 3,749 (96%) of the interviews conducted. EA selection was not stratified by district, thus 3 districts were randomly excluded from the province-wide sample. Interviews were conducted either in Portuguese or in one of the five predominant tribal languages of the province (Cisena, Elomwe, Echuabo, Cinyanja, and Emakhuwa), and data were collected using mobile cell phones. Interviewers received intensive training on the use of mobile phones for data collection prior to implementation. Satellite and census maps were used to locate the EAs. Initial plans for household selection included administering questionnaires at 15 household structures identified through a color threshold algorithm and randomly selected on the satellite map; however, the actual implementation resulted in division of the EA into four quadrants and collecting 3–4 interviews starting at the household nearest the center of each quadrant (household selection paper in preparation). In a subset of 95 randomly selected EAs, anthropometric measures of a random selection of children under 5-years residing in participating households were also included. In these EA’s, households with one or more children aged 0–12 months, one child was randomly selected for weight and height measurements. Similarly, for households with one or more children aged 13–59 months, one child was randomly selected for height and weight measurements.

Baseline survey data were collected between August and September, 2010. Fourteen teams of five female surveyors were recruited, each with prior experience in survey work. The teams were assigned by language proficiency to a specific region, working under the supervision of a regional supervisor, and were trained on general aspects of survey conduct.

Mozambique’s 2007 census served as the sampling frame. To appropriately capture *Ogumaniha*’*s* public health and development interventions without increasing the sample size and survey costs, data were collected in two phases; a concentrated sample of 2,878 households in 193 EAs in three selected focal districts (Namacurra, Alto Molócuè, and Morrumbala) (Figure 1) and a smaller sample of 871 households in 66 EA from the remaining districts. These three districts were selected because they represent 3 distinct geographical regions, and *Ogumaniha* interventions were anticipated in each, allowing future analysis of intervention impact on poverty. We provide the sample size justification online: http://globalhealth.vanderbilt.edu/manage/wpcontent/uploads/Ogumaniha_SampleSize_20100613.pdf.

### Alkire and Foster Method

Methods to identify and aggregate the poor using multiple dimensions have mathematical properties that allow for decomposition of poverty indices by subgroups or by indicators. [15] The Alkire and Foster method was used to construct three key poverty indices: headcount, average poverty gap, and adjusted headcount (called the *Ogumaniha* MPI). Application of this method is detailed elsewhere (see http://www.ophi.org.uk/research/multidimensional-poverty/how-to-apply-alkire-foster/); briefly, the steps for identification and aggregation of households include:

1. Creation of a listing of **dimensions** and corresponding **indicators** collected from the household survey. (Table 1, columns 1–2)
2. Setting **poverty lines** at values where a person is identified as deprived or non-deprived for each indicator. (Table 1, column 3)
3. Setting **weights** for contribution of each indicator to the overall metric. (Table 1, column 4)
4. Determining the poverty cut-off for **identification**. (Table 2, row 7)
5. Performing **robustness** checks of the cut-off. (Table 2)
6. Estimating across group or dimension using **aggregation**:
  - Headcount (H): proportion of households that are identified as poor.
  - Average Poverty Gap (A): weighted average deprivation experienced by poor.
  - Adjusted Headcount (called the *Ogumaniha* MPI): multiply headcount by average poverty gap to reflect the breadth of deprivations. Thus, MPI = HxA.

**Table 1:** Ogumaniha Multidimensional Poverty Index (MPI) adapted from the Oxford Poverty and Human Development Initiative (OPHI).

OPHI Model		*Ogumaniha* MPI			Districts of Alto Molócuè, Morrumbala and Namacurra
Dimension	Indicator	Deprivation cut-off (poverty line)	Weight	Deprivation	Percent of households deprived per indicator (95% CI)^1^
Education					
	Years ofSchooling	Literacy score<16 and numeracy score<5	1/6	Low literacy	14.7% (11.9, 17.5)
	ChildEnrollment	Child in household = “Yes”+age “>6” orage “<15”+attending school = “No”	1/6	School-agedchild is notattending school	17.6% (15.5, 19.7)
Health					
	ChildMortality	Fever last 30 d = “Yes”, Diarrhea last 30 d = “Yes”or Difficulty breathing last 30 d = “Yes”	1/6	Child with acuteillness	21.5% (18.5, 24.6)
	Nutrition	Household dietary diversity score<4	1/12	Low dietarydiversity	15.4% (13.1, 17.6)
		Lack of food episode during last month = “Yes”	1/12	Lack of foodepisode during lastmonth	30.4% (27.7, 33.1)
Standardof living					
	Electricity	Electricity = “No”	1/18	No electricity	95.1% (93.7, 96.5)
	Water	Water source is river = “True”, OR time to water = “>30 min”,AND mode of transport to water = “On foot”	1/18	Water source isriver or more than30 minutes awayon foot	29.7% (25.6, 33.8)
	Sanitation	Household uses latrine = “No”	1/18	No use of latrine	75.6% (72.4, 78.8)
	Flooring	Roof type = “grass/cane/leaves/straw”	1/18	Poor housingmaterial(grass roof)	92.5% (90.9, 94.0)
	Cooking Fuel	Type of fuel household uses = “Wood”	1/18	Poor cooking fuel(wood)	95.9% (94.5, 97.2)
	Assets	Sum of radio = “Yes”+television = “Yes”+bicycle = “Yes” = <1	1/18	Low assets (noradio, television,bike)	43.2% (40.7, 45.8)

1 Weighted percentages include 95% confidence intervals that incorporate the effects of stratification and clustering due to the sample design.

**Table 2:** Use of the Oxford Poverty and Human Development Initiative (OPHI) Method for Monitoring and Evaluation.

Advantages of the OPHI Method for Monitoring and Evaluation
-Expands dimension measures in critical areas including health
-Incorporates program specific detail in comparative evaluation
-Uses national comparisons for benchmarking and scale, and efficiency
-Isolates where the greatest impact is (and potentially unintended)
-Detects indirect benefits in poverty reduction from specific interventions
-Facilitates the collaboration between development policy makers who are increasingly measuring multidimensional poverty and development practitioners on the ground
-Allows for temporal and geographic comparisons

Proportions include 95% confidence intervals that incorporate the effects of stratification and clustering due to the sample design. [26] Descriptive analysis of continuous variables includes weighted estimates of median, 25th and 75th quantiles (interquartile range). Categorical variables are reported as weighted percentages, with each observation being weighted by the inverse of the household sampling probability. Tests of association by poverty ignore effects of clustering, and they include Wilcoxon rank sum (continuous) and chi-square (categorical) tests.

### Map generation

Using Esri shape files provided by INE to identify EA boundaries, a basic heat-map of poverty metrics was generated for the three focus districts. To enhance readability of the maps, ordinary kriging was used to predict poverty metrics for unsampled areas, assuming only spatial correlation. The ‘krige’ function is in the gstat package of R, which uses generalized least squares prediction with spatial covariances to produce smoothed geospatial representations of poverty. [27] R-software 2.15.1 (www.r-project.org) was employed for all data analyses; analysis scripts are available online at (http://biostat.mc.vanderbilt.edu/ArchivedAnalyses).

### Description of permanent income

**Figure 2:**
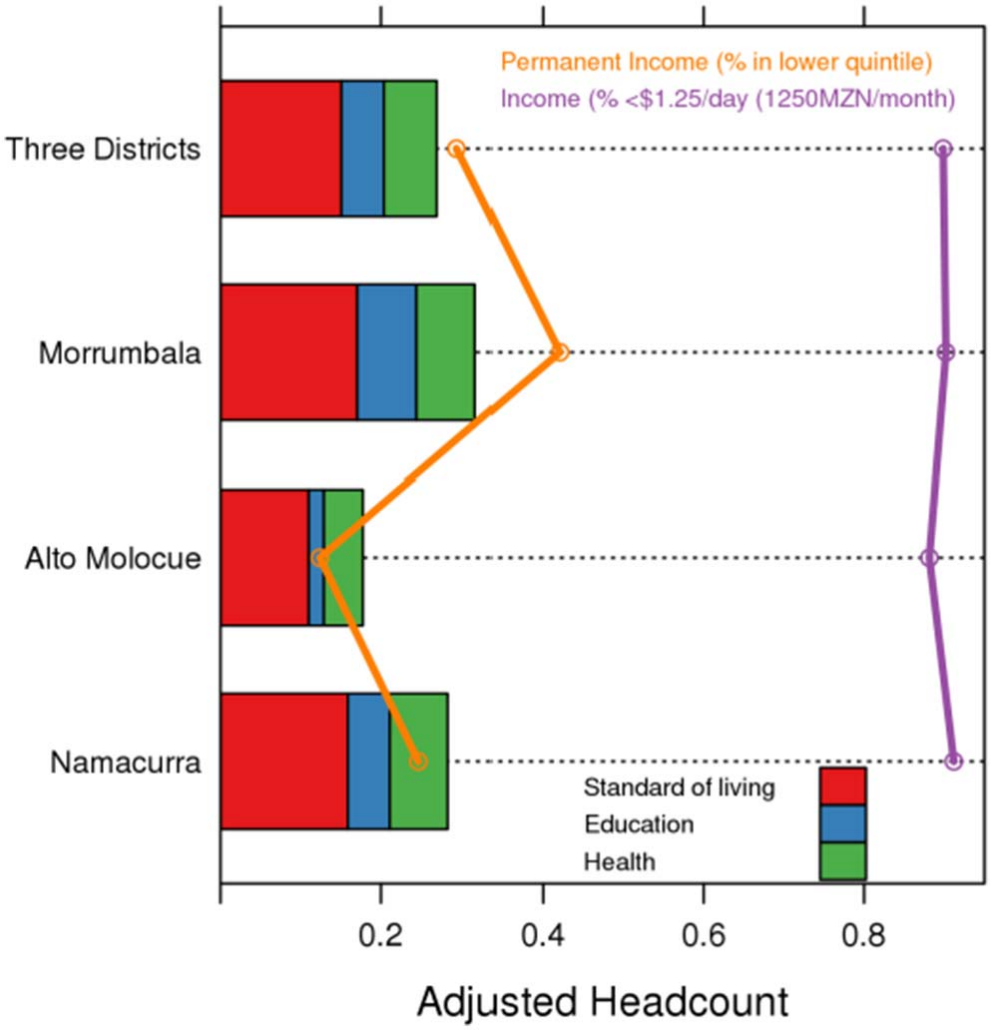
Decomposition by District and Broken Down by Dimension in the Three Focal Districts, *Ogumaniha* 2010. Legend: The adjusted headcount is decomposed by dimension for Morrumbala, Alto Molócuè, Namacurra and all three districts combined. Data that are overlaid include percent of households in the lowest quintile for permanent income wealth and % of households making less than USD$1.25/day. MZN = Metical.

The measurement of household income is particularly problematic in high poverty areas. [28] In the current sample, 49% report no monetary income whatsoever. Increasingly in economics and development, monetary income is no longer the preferred measure. Instead, a “permanent income”, [29] or wealth measure based upon ownership of selected assets is employed. Poverty stemming from lack of resources is associated with low income, but it is perhaps more closely related to low wealth. Low wealth individuals always have low income, but not all low income individuals have low wealth. In that sense, wealth and poverty are more closely related than income and poverty. We applied a measure of permanent income developed by the World Bank. [28] Briefly, a series of 37 asset and other indicator variables were used in a dichotomous hierarchical ordered probit model to derive a latent variable which denotes the permanent income of household to be incorporated into the *Ogumaniha* MPI (Figure 2, orange line) [12].

### Ethical Considerations

Participation in the household survey was completely voluntary, no incentive was provided for participation. At enrollment written informed consent was obtained. The protocol for data collection and consent forms were approved by the Mozambican National Bioethics Committee for Health (*Comité Nacional de Bioética em Saúde* [CNBS]) and the Institutional Review Board of Vanderbilt University.

## Results and Discussion

Results are presented in two sections. Section 1 describes the methodology we employed to define our metric, the *Ogumaniha* MPI, and demonstrates its usefulness to planning and evaluation of large scale public health and development interventions. Section 2 applies the *Ogumaniha* MPI to households and EAs in the three focus districts by utilizing data collected in the *Ogumaniha* baseline survey, in order to establish baseline multidimensional poverty estimates in Mozambique’s Zambézia Province.

### Section 1 - Defining the Ogumaniha Multidimensional Poverty Index (MPI)

We employ a method of monitoring and evaluation that looks at the analysis of development interventions in holistic terms, producing an aggregate metric that can be disaggregated by dimension (education, health, and living standard) and further disaggregated by subgroups (e.g., urban/rural, Portuguese proficiency). In the OPHI model, the 3 dimensions were further disaggregated into 10 indicators, weighted evenly within dimension such that each dimension had equal weight, and then a person was defined as poor if they were “deprived” in more than 33% of the indicators (Table 1). We used dimensional weighting similar to the OPHI model that would produce a comparable aggregate metric that could be used to compare across projects while incorporating national norms. Our method allows the isolation of the relative impact of specific inputs on overall well-being, and thus target areas for effective interventions.

This method goes beyond multiple measures and generates a true MPI. Specifically, it 1) uses intentionally selected dimensions of poverty to capture relevant functioning and capability deficits; 2) calculates deficit cut points based on comparative conditions; 3) applies differential weighting of deficits that contribute to poverty level; and 4) combines calculated poverty measures to produce a single metric for comparative purposes (Table 2).

An advantage of the *Ogumaniha* MPI includes its basis on survey data, which takes into account community level impact, perceptions, and buy-in. Additionally, survey data were collected utilizing a low-cost cell phone technology that reduced data entry costs and errors. The *Ogumaniha* MPI may be used for planning purposes to identify areas with high frequency of poor, intense poverty, or both. In being able to isolate not only the direct impact of a particular intervention but also the effect of inputs on other dimensions and overall well-being, this model can also serve as a useful tool in assessing return on investment and cost-benefit analyses.

### Section 2 - Identifying Poverty by Household and Enumeration Area

Building upon the OPHI MPI, we maintained the same 3 dimensions (education, health, and living standard), but defined 11 indicators rather than the 10 utilized in the OPHI model. When possible, the same OPHI indicators were used; however, some were adapted for best fit based on the questions asked in the baseline survey. Table 1 shows the OPHI Model in comparison to our adapted *Ogumaniha* MPI with the “weight” column representing the contribution of each indicator to multidimensional poverty. The rightmost column (Table 1) reports the proportion of households that are deprived in each indicator.

Of 2,878 female heads of household interviewed in the three focal districts, the median age was 32 years. The median household size was four persons and the median number of children <5 years old in the home was one. Choosing to maintain the same OPHI poverty definition cut-off, in which the “poor” must be deprived in at least 33% of the indicators, a headcount of 58.2% of our provincially representative sample met that definition and were identified as poor. The highest cut-off would identify 3.6% of households as poor whereas the lowest cut-off would identify 99.2% of households as poor. If the poor were deprived in 50% or more deprivations, then the headcount is 20% (Table 3). Approximately 96% of households lived in an EA designated as rural by INE, of which 59.5% met the criteria for being defined poor, compared to 29.3% of urban households. Looking at the relationship of poverty to the primary language spoken in the household, 26.5% of Portuguese speaking households and 58.6% of households speaking one of the other five local languages were considered poor (Table 4).

**Table 3:** Poverty Distribution of Zambézia Province, Districts of Morrumbala, Namacurra, and Alto Molócuè.

Poverty cut-off	Headcount (95% CI)
(Minimum deprivation)	(n = 2,878)
11.1%	99.2% (98.5, 99.8)
16.7%	97.7% (96.6, 98.8)
22.2%	91.4% (89.8, 93.0)
25%	77.4% (75.0, 79.8)
27.8%	75.6% (73.1, 78.2)
30.6%	64.2% (61.3, 67.1)
33.0%	58.2% (55.0, 61.4)
36.1%	52.2% (48.9, 55.5)
38.9%	44.9% (41.6, 48.3)
41.7%	35.9% (33.0, 38.8)
44.4%	33.5% (30.5, 36.4)
47.2%	25.1% (22.6, 27.7)
50%	21.0% (18.5, 23.5)
52.8%	17.0% (14.6, 19.5)
55.5%	11.5% (9.2, 13.7)
58.3%	8.9% (7.3, 10.4)
61.1%	6.9% (5.4, 8.3)
63.9%	4.7% (3.5, 5.9)
66.7%	3.6% (2.7, 4.5)

**Table 4:** Respondent and Household Characteristics by Multidimensional Poverty Status, *Ogumaniha* 2010.

Variables^a^	Non-poor	Poor	Total^d^	P-value^c^
	(n = 1196)	(n = 1682)	(n = 2878)	
Household size (n = 2878)	4 (3–6)	5 (3–6)	4 (3–6)	<0.001
Children under 5 (n = 2878)	1 (0–1)	1 (0–2)	1 (0–2)	<0.001
Age of respondent (n = 2425)	30 (23–40)	32 (25–41)	32 (24–41)	<0.001
Education (n = 2878)	2 (0–4)	0 (0–2)	0 (0–3)	<0.001
*Distance of EA from health facility (km) (n = 2878)	6.8 (3.8–10.8)	7.8 (4.5–12.2)	7.5 (4–11.7)	<0.001
Urban/rural (n = 2878)				<0.001
Rural	40.5%	59.5%	95.7%	
Urban	70.7%	29.3%	4.3%	
Length of residency (years) (n = 2785)	6 (3–18)	6 (3–15)	6 (3–17)	0.997
Primary language of household (n = 2873)				<0.001
Cinyanja	23.2%	76.8%	0.8%	
Cisena	33.7%	66.3%	44.3%	
Echuabo	39.8%	60.2%	25.6%	
Elomwe	55.7%	44.3%	27.6%	
Emakhuwa	45.4%	54.6%	0.3%	
Portuguese	73.5%	26.5%	1.3%	
Respondent understands Portuguese (n = 2876)	54.7%	45.3%	27.5%	<0.001
Marital status (n = 2878)				0.035
Married/Common Law	42.8%	57.2%	72.9%	
Divorced/Separated	29.5%	70.5%	3.4%	
Single	43.0%	57.0%	17.5%	
Widowed	34.0%	66.0%	6.2%	
Religion (n = 2598)				<0.001
Catholic	48.0%	52.0%	45.7%	
Protestant	41.2%	58.8%	10.7%	
Evangelical and Pentecostal	39.6%	60.4%	14.3%	
Other Christian^b^	37.1%	62.9%	4.5%	
Muslim	41.1%	58.9%	8.5%	
Non-Christian Eastern	37.8%	62.2%	4.3%	
Other^b^	34.2%	65.8%	11.9%	
**Monthly household income, Meticais (n = 2691)	150 (0–500)	0 (0–300)	150 (0–500)	<0.001
No household income (n = 2691)	33.4%	66.6%	48.8%	<0.001
Household member has a farm (n = 2856)	41.6%	58.4%	90.7%	0.408
Permanent income (n = 2878)	0.6 (0.3–0.8)	0.3 (0–0.5)	0.4 (0.1–0.7)	<0.001
Ever accessed health facility (n = 2878)	45.4%	54.6%	57.4%	<0.001
Ever accessed pharmacy (n = 2878)	46.5%	53.5%	18.1%	0.004
Ever accessed traditional healer (n = 2878)	43.8%	56.2%	39.7%	0.579
***Ever accessed VCT (n = 2878)	55.1%	44.9%	10.7%	<0.001
****Accessed ANC last pregnancy (n = 2457)	44.7%	55.3%	41.8%	0.001

a Continuous variables are reported as weighted estimates of median (interquartile range] and categorical variables are reported as weighted percentages, with each observation being weighted by the inverse of the household sampling probability.

b ‘Other Christian’ includes LDS Mormon and Jehovah’s Witness. ‘Other’ includes Spiritual, Traditional Religions, and Agnostic or Atheist.

c Tests of associations (continuous) include Wilcoxon rank sum (continuous) and chi-squared test (categorical).

d All percentages in the cross tabulations are row percentages. The final column presents column (overall) percentages.

*EA = enumeration area.

**Approximate exchange rate as of October 2013: $1 USD = 30 Meticais.

***VCT = voluntary counseling and testing (for HIV).

****ANC = antenatal clinic.

Once identified as poor, if the intensity of deprivation increases for a household (say from 33% to 60% of weighted indicators), the headcount remains unchanged. As such, robust models must be able to adjust for changes in both headcount and the average deprivation; this MPI is called adjusted headcount. Using the 33% deprivation cut-off from the OPHI method, we estimated a headcount of 58.2% of the three focal districts sample to be poor; among these, households experienced an average deprivation of 46%; thus the adjusted headcount ( = 0.58×0.46) is 0.27 (95% CI: 0.25–0.29). The headcounts for the three focal districts (Morrumbala, Namacurra, Alto Molócuè) plus all three combined are 0.67, 0.60, 0.42, and 0.58, respectively. The average deprivation for the three focal districts, plus all three combined, are 0.48, 0.47, 0.42, and 0.46. Analyzed separately, Morrumbala has the greatest breadth of poverty with an index of 0.32 (0.29–0.34), followed by Namacurra at 0.28 (0.26–0.31), and the least deprived being Alto Molócuè with an index of 0.18 (0.15–0.21). Additionally, the *Ogumaniha* MPI can be further broken down revealing the weight of each dimension’s contribution to that index. In all three districts, living standard was the most dominant dimension deprived, followed by health, and then education (Figure 2).

**Figure 3:**
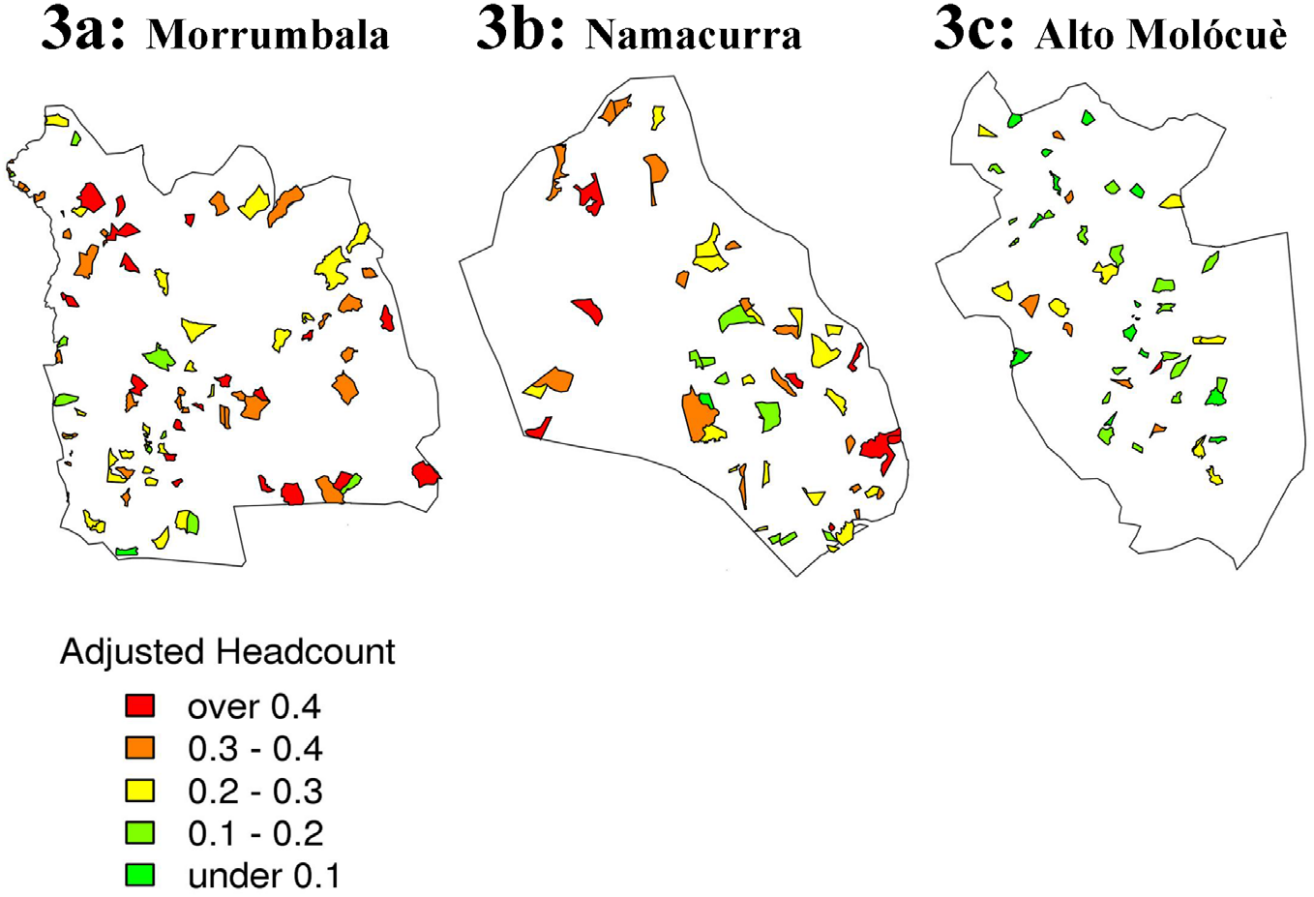
Enumeration Area Distribution in Three Focus District by Adjusted Headcount: Morrumbala, Namacurra and Alto Molócuè. *Enumeration area representations of poverty by adjusted headcount with green being less deprived and red.

**Figure 4:**
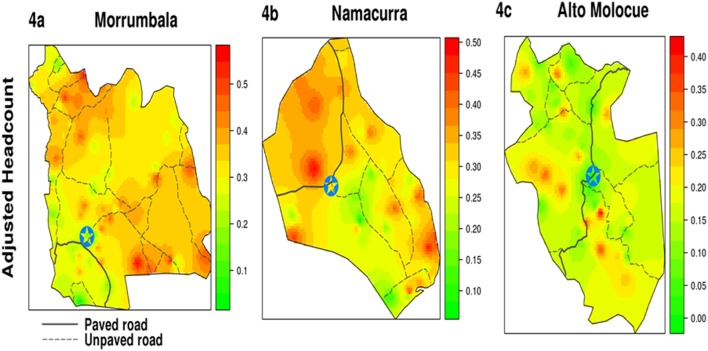
Smoothed Heat Map of Three Focus Districts: Morrumbala, Namacurra, and Alto Molócuè. *Figures 4a, 4b, and 4c show heat map geographical representations of poverty by adjusted headcount with green being less deprived and red most deprived. (Circled Star represents location of district capital).

Following calculation of the *Ogumaniha* MPI for each of the 193 EAs in three focal districts, geospatial maps were created that allow for visualization of the dispersion of EAs throughout the three districts (Figure 3) and provide smoothed estimates of poverty at baseline (Figure 4). The mapping of poverty deprivation at baseline provides program implementers a visual needs assessment that characterizes the geographic differences of each target district and aids in prioritizing program planning. Additionally, going forward we can overlay maps depicting the location and intensity of the various *Ogumaniha* sector interventions in the target districts (which were not uniformly implemented) and evaluate the impact of those interventions, or the combination of interventions. This is possible for measured outcomes in the specific individual-level locations in which interventions were implemented and also for a broader area-level evaluation or “neighborhood effect” that interventions may or may not have on nearby locales and/or the district as a whole.

In Morrumbala (Figure 4a), there are two geographic areas of high poverty deprivation, one in the extreme northwest of the district and the other in the southeastern region. Areas of least poverty deprivation, tend to focus around the main transportation corridor in the south, around the district capital and extends to the northeast through the center of the district. The area of high poverty deprivation in the northeast is fairly isolated, and difficult to reach (can take up to 5 hours to reach by car from the district capital). Morrumbala suffers from flooding of the Zambezi and Chire rivers each year. Flood zones are located in the southwest region and extending north along the western border of the district.

In Namacurra (Figure 4b), the largest geographic area of high poverty deprivation is in the northwest side of the district with pockets of high poverty deprivation distributed throughout the southeast. The southern parts of Namacurra can be difficult to reach with roads that flood, cutting them off from the rest of the district during the rainy season. However this area has some of the more fertile farm-land. Furthermore, the southernmost tip of Namacurra is coastal (one of only a handful of natural deep water ports on the eastern African coastline). This port is slated for large investments in infrastructure over the next 5–10 years, and may become an economic hub for the north-central provinces of Mozambique.

Alto Molócuè (Figures 4c) is one of the more economically developed districts with several commercial factories. The national highway cuts across the district and connects Alto Molócuè with commercial centers in the south and Nampula Province to the north. Pockets of high deprivation tend to be located in areas farthest away from transportation corridors.

## Conclusions

We seek innovative approaches to conducting evaluation research in public health. [30] Baseline assessments of poverty that are more valid can improve our program planning as well as evaluation of interventions. Advancement in evaluation is increasingly critical due to the pressures created by the burdens of disease in the developing world, and even more so by the increasing complexity of interventions. In developing the measurement and evaluation tools reported here we employ the most current understanding of the crucial interdependency between conditions of poverty and both the need for and effectiveness of public health interventions. However, significant obstacles still limit the potential utility of this new approach to public health program evaluation. While measures taking multidimensionality into account have already been adopted by the Mozambican government, its meaning and use remains relatively unfamiliar. In our sample, relatively few households sampled came from urban areas as the make-up of Zambézia Province is predominantly rural. In our context we feel the MPI measure accurately captures a relevant view of the condition of poverty across the province. However, a limitation to this approach is that if applied to a predominantly urban area, the indicators used to assess poverty may overestimate households as non-deprived based on a lack of dirt floor for example, despite the fact that they may live in a poorly quality structures with crowded conditions and limited space. As a result, analysis must take into account context for what is considered deprived or not. Additionally, the logistics, manpower, and costs of implementing such a large population-based survey at program’s beginning and end may limit the accessibility of this approach to only governments, donor agencies, and/or larger multi-sector public health development projects whose outcomes of interest are not isolated to the individual sector specific program indicators.

In this paper, we have reported the estimated baseline MPI for Zambézia Province at initiation of a large scale USAID funded, 5-year multi-sector grant. We believe that our estimates of poverty are a conservative minimum, as our results show that greater than half of our representative sample is poor (based on the 33% deprivation standard), and one-fifth or more are deprived in 50% of indicators. We found that living standard was the dominant dimension of poverty, followed by health and then education. By avoiding a unidimensional income measure, which in the Zambézia context classifies >90% of households as poor based on the generic < $1.25/day standard (Figure 2), the *Ogumaniha* MPI reveals a more nuanced and differentiated view of the conditions of poverty in an area that frequently employs non-monetary economic structures and is reliant on subsistence farming and fishing. This will assist in setting priorities for development in an area of near-universal poverty, as the neediest persons and regions can be differentiated from the less needy.

The implications of this work apply to both program planning and research. The integration of multidimensional poverty measures into planning and implementation design and the use of geospatial visualization of the relationships between interventions and distinct conditions of poverty at baseline have great utility and added value to the field of public health. Geospatial mapping, for instance, can dramatize variations of poverty in microenvironments, helping set program intervention priorities. Key to meeting the twin demands of greater impact and greater efficiency for public health interventions is recognizing and responding to the challenges created by the interdependencies between public health and other dimensions of poverty. Such interdependencies are rarely incorporated into program evaluation, thus leaving public health implementers and researchers struggling to understand both the positive and negative outcomes of interventions.

The MPI technique allows public health professionals and agencies to better target their resources by accounting for variation. With respect to research, we demonstrate how a robust multidimensional measurement can explore the linkages between poverty and health differentials in a population and derive useful inferences for further evidence-based planning. When repeated in Zambézia at the end of the 5-year program, evaluators will be permitted to not only observe the relative impact of *Ogumaniha*’*s* intervention intensity, duration, and composition, but also analyze factors that drive or inhibit direct effectiveness and interaction effects, at both the micro/household level as well as a more macro/provincial level. This work demonstrates the distinctive and highly valuable insights that are created by applying a multidimensional measure of poverty to the evaluation of a complex, large scale public health intervention such as the *Ogumaniha*-SCIP project.

## References

[1] The United Nations Development Agenda: Development for all. Available: http://www.un.org/esa///devagenda/UNDA_BW5_Final.pdf. Accessed 2014 Sep 5.

[2] Fanzo JC, Pronyk PM (2011) A review of global progress toward the Millennium Development Goal 1 Hunger Target. Food Nutr Bull 32: 144–158. 22164975 10.1177/156482651103200207

[3] Dodd R, Cassels A (2006) Health, development and the Millennium Development Goals. Ann Trop Med Parasitol 100: 379–387. 16899143 10.1179/136485906X97471

[4] Gaffikin L, Ashley J, Blumenthal PD (2007) Poverty reduction and Millennium Development Goals: recognizing population, health, and environment linkages in rural Madagascar. Medscape Gen Med 9: 17. PMC2234285 18311367

[5] Anand S, Sen A Concepts of human development and poverty: A multidimensional perspective. Available: http://clasarchive.berkeley.edu/Academics/courses/center/fall2007/sehnbruch/UNDP%20Anand%20and%20Sen%20Concepts%20of%20HD%201997.pdf. Accessed 2014 Sep 5.

[6] Saith A (2011) Inequality, imbalance, instability: reflections on a structural crisis. Dev Change 42: 70–86.

[7] Sen A (2000) A decade of human development. J Hum Dev 1: 17–23.

[8] International Human Development Indicators - UNDP: Mozambique. Available: http://hdr.undp.org/en/countries/profiles/MOZ. Accessed 2014 Sep 5.

[9] Bourguignon F, Chakravarty SR (2003) The measurement of multidimensional poverty. Journal of Economic Inequality 1: 25–49.

[10] Guedes GR, Brondizio ES, Barbieri AF, Anne R, Penna-Firme R, et al (2012) Poverty and inequality in the rural Brazilian Amazon: A multidimensional approach. Hum Ecol 40: 41–57. 10.1007/s10745-011-9444-5 PMC3426830 22927705

[11] Deutsch J, Silber J (2005) Measuring multidimensional poverty: An empirical comparison of various approaches. Rev Income Wealth 51: 145–174.

[12] Victor B, Fischer EF, Cooil B, Vergara A, Mukolo A, et al (2013) Frustrated freedom: The effects of agency and wealth on wellbeing in rural Mozambique. World Dev 47: 30–41. 25125791 10.1016/j.worlddev.2013.02.005 PMC4128575

[13] The multidimensional poverty assessment tool (MPAT): A new approach for measuring rural poverty. Available: http://www.ifad.org/mpat/. Accessed 2014 Sep 5.

[14] Alkire S, Santos ME (2011) Acute multidimensional poverty: A new index for developing countries (2011). In Proceedings of the German Development Economics Conference, Berlin, No. 3.

[15] Alkire S, Foster J (2011) Counting and multidimensional poverty measurement. J Public Econ 95: 476–487.

[16] Sagar AD, Najam A (1998) The human development index: a critical review. Ecol Econ 25: 249–264.

[17] Country Health Profile of Mozambique - WHO Regional Office for Africa. Available: http://www.afro.who.int/en/mozambique/country-health-profile.html. Accessed 2014 Sep 5.

[18] WHO World Health Statistics 2011. Available: http://www.who.int/whosis/whostat/2011/en/index.html. Accessed 2014 Sep 5.

[19] INSIDA 2009, Inquérito Nacional de Prevalência, Riscos Comportamentais e Informação sobre o HIV e SIDA em Moçambique. Available: http://www.measuredhs.com/pubs/pdf/AIS8/AIS8.pdf. Accessed 2014 Sep 5.

[20] Multiple Indicator Cluster Survey (2008). Available: http://www.unicef.org/mozambique/MICS_Summary_English_201009.pdf. Accessed 2014 Sep 5.

[21] Moon TD, Burlison JR, Sidat M, Pires P, Silva W, et al (2010) Lessons learned while implementing an HIV/AIDS care and treatment program in rural Mozambique. Retrovirology: Research and Treatment 3: 1–14. 25097450 10.4137/RRT.S4613 PMC4119752

[22] Moon TD, Burlison JR, Blevins M, Shepherd BE, Baptista A, et al (2011) Enrolment and programmatic trends and predictors of antiretroviral therapy initiation from president’s emergency plan for AIDS Relief (PEPFAR)-supported public HIV care and treatment sites in rural Mozambique. Int J STD AIDS 22: 621–627. 22096045 10.1258/ijsa.2011.010442

[23] Cook RE, Ciampa PJ, Sidat M, Blevins M, Burlison J, et al (2011) Predictors of successful early infant diagnosis of HIV in a rural district hospital in Zambézia, Mozambique. J Acquir Immune Defic Syndr 56: e104–109. 21266912 10.1097/QAI.0b013e318207a535 PMC3073723

[24] Ciampa PJ, Vaz LME, Blevins M, Sidat M, Rothman RL, et al (2012) The association among literacy, numeracy, HIV knowledge and health-seeking behavior: a population-based survey of women in rural Mozambique. PloS One 7: e39391. 22745747 10.1371/journal.pone.0039391 PMC3382184

[25] Ministério da Saúde-Moçambique: HIV/SIDA. Available: http://www.misau.gov.mz/index.php/hiv-sida. Accessed 2014 Sep 5.

[26] Lumley T (2004) Analysis of complex survey samples. Journal of Statistical Software 9(1): 1–19.

[27] Pebesma EJ (2004) Multivariable geostatistics in S: the gstat package. Comput Geosci 30: 683–691.

[28] Ferguson J (1999) *Expectations of modernity: Myths and meanings of urban life on the Zambian copperbelt*. Berkeley: University of California Press.

[29] Friedman M (1957) *A Theory of the Consumption Function*, Princeton: Princeton University Press.

[30] Editorial (2010) Evaluation: the top priority for global health. The Lancet 375: 526. 10.1016/S0140-6736(10)60056-6 20079530

